# Safety, biodistribution and radiation dosimetry of the Arg-Gly-Asp peptide ^99m^Tc-maraciclatide in healthy volunteers

**DOI:** 10.1097/MNM.0000000000001814

**Published:** 2024-02-05

**Authors:** Tatjana Gibbons, Alan Perkins, Jon Barnett

**Affiliations:** a Nuffield Department of Women's and Reproductive Health, University of Oxford, John Radcliffe Hospital, Oxford,; b Radiological Sciences, School of Medicine, University of Nottingham, Nottingham and; c Serac Healthcare Ltd., London, UK

**Keywords:** ^99m^Tc, maraciclatide, Arg-Gly-Asp peptide, dosimetry, SPECT-CT, whole, body biodistribution

## Abstract

**Background:**

^99m^Tc-Maraciclatide is a radiolabelled RGD (Arg-Gly-Asp) peptide that binds with high affinity to α_v_β_3_ and α_v_β_5_ integrins, common receptors upregulated in disease states involving angiogenesis and inflammation. As such, it holds promise as a novel diagnostic imaging agent for a range of pathological conditions. The present study provides the safety, biodistribution and radiation dosimetry of ^99m^Tc-maraciclatide in healthy volunteers.

**Methods:**

A phase 1, randomised, placebo-controlled study assessed the safety, biodistribution and radiation dosimetry of ^99m^Tc-maraciclatide in healthy volunteers. Participants were randomised into three groups receiving ^99m^Tc-maraciclatide and three chemical amounts of maraciclatide in an escalating dose protocol. Eight participants in each group received the required amount of maraciclatide via intravenous injection, with the remaining two receiving a placebo. Biodistribution was assessed by acquiring scintigraphic images at time points up to 24 h after a bolus injection of ^99m^Tc-maraciclatide. ^99m^Tc-maraciclatide activity in plasma and urine was measured up to 7 days post-administration.

**Results:**

^99m^Tc-maraciclatide was safe and well tolerated, with no serious adverse events reported. Initial uptakes of ^99m^Tc were highest in the gastrointestinal tract (20%), liver (15%), and lungs (9%). Similarly, the regions with the highest normalised cumulated activities were the contents of the urinary bladder and voided urine (3.4 ± 0.4 MBq*h/MBq), the combined walls of the small intestine and upper and lower large intestine (0.9 ± 0.2 MBq*h/MBq), liver (0.8 ± 0.2 MBq*h/MBq), lung (0.4 ± 0.1 MBq*h/MBq). The main route of ^99m^Tc excretion was renal (55%), with a systemic urinary clearance of approximately 6.7 ml/min/kg. The pharmacokinetic analysis gave a mean apparent terminal elimination half-life of the unlabelled molecular maraciclatide of approximately 1 h, independent of dose. The mean ED per unit injected activity was 7.8 ± 0.8 µSv/MBq.

**Conclusion:**

^99m^Tc-maraciclatide is a safe radiopharmaceutical formulation with a dosimetry profile similar to other ^99m^Tc-based imaging agents.

## Introduction

Integrins are transmembrane proteins that are essential in various cellular processes, such as cell adhesion, proliferation, and migration [1]. There are 24 known integrins which differ in their alpha and beta subunit composition [1,2]. Angiogenesis, the formation of new blood vessels from pre-existing ones, is one of the most well-known natural biological processes regulated by integrins. In adults, angiogenesis occurs during wound healing, muscle growth, and in diseases such as arthritis, endometriosis, and neoplasia [1–3]. One of the most extensively studied integrins involved in angiogenesis is α_v_β_3_. α_v_β_3_ integrin expression is a valuable molecular target since it is upregulated in diseased states but relatively quiescent in healthy tissues. Thus, detecting α_v_β_3_ holds potential for diagnostic advances, monitoring therapeutic responses to therapeutic agents, and advancing our understanding of disease mechanisms [4–7].

**Fig. 1:**
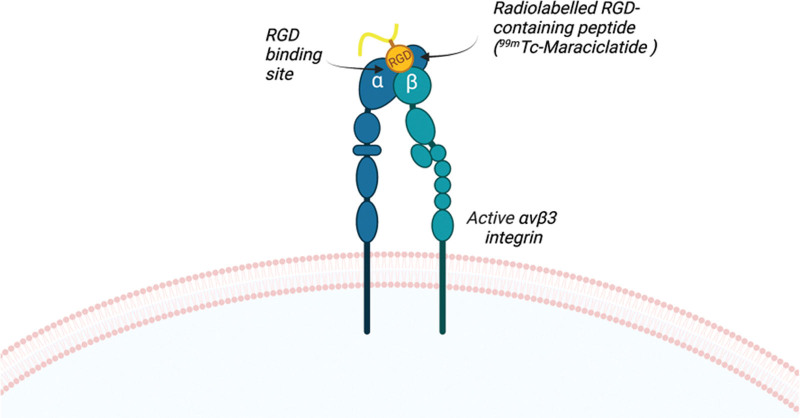
Diagram of αvβ3 integrin in the active conformation, bound to ^99m^Tc-maraciclatide via the RGD component (created on Biorender).

Maraciclatide (previously known as NC100692) is a synthetic molecule that specifically binds via the Arginine-Glycine-Aspartic (RGD) amino-acid motif to α_v_β_3/5_ integrin receptors (Fig. 1). The molecule includes a chelator for radiolabelling with the readily available imaging radionuclide, technetium [^99m^Tc], enabling visualisation of distribution and accumulation of the tracer after intravenous injection by gamma scintigraphy or SPECT-CT. The diagnostic application of ^99m^Tc-maraciclatide has been applied to human and animal research in detecting bone metastasis, malignant breast tumours, atherosclerotic plaques, rheumatoid arthritis, and peripheral vascular disease [5–10]. ^99m^Tc-maraciclatide has shown particular promise in detecting synovitis in rheumatoid arthritis, resulting in further studies in this and other areas, such as endometriosis, which share similar molecular and cellular processes and genetic associations [7,11–13]. As such, it is essential to understand the radiopharmaceutical safety and biodistribution through human dosimetry studies.

## Materials and methods

In 2003, a phase 1, placebo-controlled, observer-blinded, randomised, single ascending-dose study was performed. The study aimed to assess the safety of three intravenous levels of maraciclatide and the biodistribution and radiation dosimetry of ^99m^Tc-maraciclatide in healthy volunteers, assessing the potential of ^99m^Tc-maraciclatide as a radiopharmaceutical for diagnostic imaging.

### Radiopharmaceutical preparation

Maraciclatide is a chelate-peptide conjugate containing an Arginine-Glycine-Aspartic (RGD) amino-acid motif in such a configuration that it specifically binds with nanomolar affinity to the integrin receptor α_v_β_3_. Radiolabelling of the product was performed under aseptic conditions in a phase 1 unit. Maraciclatide (lot FFA082/072-205-2) was originally supplied as lyophilised kits (GE Healthcare, Oslo, Norway). Each vial contained approximately 44 nmol maraciclatide (molecular weight 1697). The lyophilised kits were stored at −20°C. ^99m^Tc labelling was performed by reconstitution with ^99m^Tc-sodium pertechnetate with incubation at room temperature for 20 min. The radiolabelled product could be used for up to 3 h post-reconstitution. The radiochemical purity of the product was tested using HPLC and instant thin-layer chromatography, showing the kit could maintain stable radiochemical purity above 85% for over 4 h (Table 1). All the preparations used in the study had a radiochemical purity ≥85% and reduced hydrolysed technetium (RHT) content below 4%.

**Table 1:** Radiochemical stability data from freeze-dried kits (lot FFA082/072-205-2) stored for 3 months at 2–8 °C

Storage time (months)	Radiochemical purity (RCP) 20 min (%)	Radiochemical purity (RCP) 4 h (%)	Reduced hydrolysed technetium (RHT) (%)	^99m^Tc activity added (GBq)
**0**	93.0	87.5	<1	2.20
**2**	92.5	85.5	<1	2.03
**3**	92.5	88.0	<1	2.05

In-vivo stability of maraciclatide has previously been demonstrated from urine samples taken from participants in this study using reversed-phase liquid chromatography coupled with an ion-trap mass spectrometer (LC-MS) in order to estimate the amount of intact maraciclatide and any metabolites excreted in the highest dose group (150 micrograms maraciclatide) [14]. Only intact maraciclatide was observed in the urine samples, and no metabolites were detected.

Three chemical dose levels (the chemical amount of maraciclatide administered) were studied, and the way this was achieved for each group is presented in Table 2. The relevant factor increased the other components of the formulation.

**Table 2:** The chemical amount of maraciclatide administered to each group, expressed in terms of equivalent vials administered

Chemical dose group	Reconstitution volume per vial (ml)	Number of vials used	Total bulk volume (ml)	Injection volume (ml)	Maraciclatide administered (μg)	Maraciclatide as % of 1 kit vial
1	8	1 vial	8	1.6	15	20%
2	2	2 vials	4	2.0	75	100%
3	2	3 vials	6	4.0	150	200%

The upper limit of ^99m^Tc activity administered to healthy participants in the present study was 200 MBq (5.4 mCi) to minimise the radioactive dose. In subsequent clinical imaging studies, 740–1100 MBq (20.0–29.7 mCi) has been routinely used.

### Participants

Approval for this study was obtained from the Quorn Research Review Committee (Independent Ethics Committee (IEC)) according to UK regulations, and informed consent was received from all participants.

Thirty-one participants (19 males and 12 females) were recruited for this study (Table 3), aged 22–64. There were no significant differences in baseline characteristics between groups. The inclusion criteria included ages 18–65 with a normal medical history, physical exam, vital signs, laboratory tests, ECG, and drug screen. The females included in the study were post-menopausal or surgically sterilised with a negative urine pregnancy test. The exclusion criteria included those who were pregnant, lactating or had received another investigational product within 3 months.

**Table 3:** Summary of subject numbers recruited, administered and evaluated

Subjects recruited	Subjects receiving ^99m^Tc-maraciclatide	Subjects receiving ^99m^Tc-maraciclatide evaluated for biodistribution and dosimetry	Subjects receiving ^99m^Tc-maraciclatide evaluated for safety	Subjects receiving placebo and evaluated for safety
N = 31	N = 25 8 dose group 1 8 dose group 2 8 dose group 3 1 incorrect chemical dose	N = 25 8 dose group 1 8 dose group 2 8 dose group 3 1 incorrect chemical dose	N = 24 8 dose group 1 8 dose group 2 8 dose group 3	N = 6 2 dose group 1 2 dose group 2 2 dose group 3

The study planned to recruit thirty participants divided into three dose groups. Two of the 10 participants were randomly assigned to receive a saline placebo in each dose group. The saline placebo was administered in the same manner as the ^99m^Tc-maraciclatide, including using a lead-shielded syringe and receiving a scintigraphic scan at the same time intervals. The safety assessors were also blinded to administering a placebo or ^99m^Tc-maraciclatide. The scintigraphic camera monitor was shielded to prevent images from being seen, and local radioactivity monitors were switched off to remove any audio signal. Within each group, at least two females and two males in each dose group received ^99m^Tc-maraciclatide.

### Administration

^99m^Tc-Maraciclatide at the three different maraciclatide chemical dose levels was administered to 25 subjects as a single intravenous administration (Tables 2 and 3). A non-radioactive placebo (saline) was administered to 6 subjects as a single intravenous administration as an equivalent volume to each dose group.

Subjects received ^99m^Tc-maraciclatide under the direct supervision of study personnel. An intravenous line was established, with an intravenous catheter placed into an appropriate vein. Subjects received an intravenous administration of ^99m^Tc-maraciclatide at 2–4 ml per second. After administration, the line was flushed with 5 ml of Sodium Chloride (0.9% w/v). The administration site was evaluated pre- and post-administration for any reaction or extravasation.

One participant in the maraciclatide 75 μg group received a lower chemical dose than intended (47 μg), so an additional participant was enrolled. Data for the participant who received the incorrect chemical dose were excluded from the safety and pharmacokinetics analysis but included in the biodistribution and dosimetry calculations (Table 3).

### Safety

Safety data on vital signs (heart rate, blood pressure, temperature, and oxygen saturation), electrocardiograms (ECG), laboratory parameters and physical examinations (general appearance, lung, abdominal, cardiovascular, and neurological exam by a qualified clinician) were measured prior to administration, and at multiple time points up to 7 days following administration. Based on clinical assessment, new abnormal or worsening clinical findings were defined as a change from the participant’s normal baseline to abnormal. All abnormal changes were recorded as adverse events (AEs).

Safety assessments were performed by an observer blinded to the administration of the placebo and ^99m^Tc-maraciclatide. A thorough review of safety data was performed before the decision was made to escalate to the higher dose in participants.

### Image acquisition and in-vivo activity measurement

On the day of administration before the administration of ^99m^Tc-maraciclatide, a transmission scan was obtained. The counts acquired from a flood or line source with and without the participant on the imaging couch were utilised to calculate body attenuation. Following intravenous administration of ^99m^Tc-maraciclatide, whole-body gamma scintigraphy was performed at 5 min post-administration up to 24 h as described below (section 2.6). Additional anterior and posterior planar images of the chest (45 min and 1 h 45 min, post-administration) and the pelvis (1 h 15 min and 2 h post-administration) were obtained for each participant.

Regions of interest (ROIs) were selected from organs and tissues on the whole-body scintigraphic images showing significant activity uptake. ^99m^Tc activity in ROIs was calculated using the methodology described in Medical Internal Radiation Dose (MIRD) Pamphlet No. 16 [15].

Whole body, chest and pelvic scintigraphic images were assessed by recording distinct areas of focal uptake of radioactivity to determine whether images from healthy volunteers contain foci that could be mistaken as lesions. Focal uptake included any local accumulation of radioactivity seen in the images, excluding expected organ uptake. In contrast, the radiation dosimetry report presented whole organ uptake data. Whole-body scans were assessed by recording the number of distinct areas with focal uptake (with ‘0’ recorded if none were seen).

### Measurement of ex-vivo activity

To calculate ^99m^Tc-maraciclatide ex-vivo activity, blood, plasma, urine, and faeces were collected at various time points after administration. Blood and plasma samples were collected at 10 min, 30 min, 1 h, 2 h, 4 h, 8 h, 24 h, 48 h, 72 h, and 7 days post-administration. Concentrations of maraciclatide in plasma were assayed using the LC-MS method [14] (LOQ was 0.5 ng/ml). Urine was also collected at the same time points (if possible) up to 24 h post-administration. Faecal samples were collected as voided for up to 24 h post-administration. Assayed samples for ^99m^Tc-maraciclatide content were then measured using a gamma counter.

### Biodistribution and radiation dosimetry

The biodistribution of ^99m^Tc was determined by whole-body conjugate view imaging in conjunction with extracorporeal activity measurements in whole blood, plasma, voided urine, and faeces, as described above. Simultaneous anterior and posterior whole-body images were acquired beginning nominally at 5 min, 15 min, 30 min, 60 min, 90 min, 3 h, 6 h and 12 h post-administration. A final anterior and posterior image pair was acquired at a ninth time-point between 21 and 24 h post-administration. Quantitative analysis of the incorporated data at multiple time points yielded biodistribution data consisting of activity in 10 specified organs or tissues [brain, salivary glands, thyroid gland, lungs, heart, spleen, liver, gallbladder, gastrointestinal (GI) tract and urinary bladder]. The remaining ^99m^Tc activity in other tissues was grouped within a ‘remaining tissues’ category. For each organ or tissue of interest, the ^99m^Tc activity was decay-corrected to the administration time and normalised to the injected activity. The temporal variation of this normalised and decay-corrected activity was then fit by one of several analytical functions, which was then integrated to yield the cumulated activity for that organ normalised to the injected activity.

The mean absorbed doses per unit injected activity to 24 target organs were evaluated from this matrix of normalised cumulated activity values, using the MIRD schema [15], for a 70-kg adult human. The cumulated activity in the urinary bladder contents and voided urine was calculated using a dynamic urinary bladder model for a 3.5-h voiding interval. Cumulative activities in the GI tract were calculated using a dynamic model that assumed that activity enters the GI tract solely through hepatobiliary drainage. At early times (up to 90 min) post-administration, the GI tract exhibited a diffuse uptake of ^99m^Tc. At later times, it was possible to identify the ^99m^Tc associated with the GI tract as predominantly that in the luminal contents. The overall time course of ^99m^Tc activity in a ROI set over the GI tract was typically that of an initial rapid decrease which then slowed to approach the amount present in the luminal contents. This observation was considered to be due to an initial rapid uptake and subsequent tissue clearance of ^99m^Tc from the GI tract walls combined with the slow introduction of ^99m^Tc into the GI contents via hepatobiliary transport. The activity in the luminal contents was isolated by first modelling the GI tract wall tissue clearance by the renormalised time course of activity measured in a ROI set over a region of soft tissue (the quadriceps femoris in one leg). This modelled GI tract wall tissue clearance was then subtracted from the measured activity in the GI tract wall and contents to yield the activity in the luminal contents, which displayed the expected asymptotic increase. The ED per unit injected activity was subsequently calculated from the ensemble of organ-absorbed dose values.

## Results

Unless otherwise specified, all values presented are the mean with uncertainties given as one SD. All activities have been decay-corrected to the time of administration and are normalised to the injected activity.

### Participants

All enrolled participants completed the study (24 received the protocol-stipulated quantity of maraciclatide, 1 received an incorrect amount of maraciclatide, and 6 received a placebo), and their data were analysed (Tables 3–5). Female participants were enrolled into all dose groups and into the placebo groups. No significant differences were seen between the dose groups/placebo except for the mean age of the participants in the 150 μg dose group. This was due to the inclusion of more male participants, who were generally younger than the females in the study.

**Table 4:** Participant characteristics recorded at baseline

Characteristic		Maraciclatide 15 μg N = 8	Maraciclatide 75 μg N = 8	Maraciclatide 150 μg N = 8	Placebo N = 6
Age	Mean (SD)	49.6 (12.1)	49.0 (8.4)	34.3 (11.7)	47.8 (12.6)
	Range	29–64	43–64	22–56	25–61
Height	Mean (SD)	169.8 (6.5)	168.1 (7.7)	169.8 (9.7)	170.2 (9.4)
	Range	157–177	156–177	152–180	153–180
Weight	Mean (SD)	71.9 (9.1)	73.6 (7.7)	75.9 (11.4)	69.7 (9.4)
	Range	69–90	66–85	58–91	58–85
BMI	Mean (SD)	24.9 (2.7)	26.0 (1.5)	26.3 (3.2)	24.1 (3.0)
	Range	21–29	24–28	21–33	20–28
Gender	Male	4	5	6	4
	Female	4	3	2	2
Race	Black	1	0	0	0
	Caucasian	7	8	8	6

**Table 5:** Participant concurrent medications or therapies taken within 14 days before and up to the end of the observation period

Primary WHO class	Secondary WHO class	Maraciclatide 15 μg N = 8 N (%)	Maraciclatide 75 μg N = 8 N (%)	Maraciclatide 150 μg N = 8 N (%)	Placebo N = 6 N (%)
Cardiovascular	Vasoprotectives	0 (0)	0 (0)	0 (0)	1 (16.7)
Dermatology	Antibiotics and chemotherapeutics for dermatology	0 (0)	0 (0)	1 (12.5)	0 (0)
	Emollients	1 (12.5)	0 (0)	0 (0)	0 (0)
Genito urinary system and sex hormones	Sex hormones	1 (12.5)	1 (12.5)	0 (0)	0 (0)
Nervous system	Analgesics	0 (0)	1 (12.5)	1 (12.5)	0 (0)
Total number of medications other than the investigational product		2 (25.0)	2 (25.0)	2 (25.0)	0 (0)

### Administration

The radiochemical purity was above 92%, and the RHT was not above 1.7% (Table 6) for all subjects.

**Table 6:** Summary of the radiochemical purity of ^99m^Tc-maraciclatide, quantity of RHT, the injected activity and the volume administered

Dosing parameter		Maraciclatide 15 μg N = 8	Maraciclatide 75 μg N = 8	Maraciclatide 150 μg N = 8
Radiochemical purity (%)	Mean (SD)	95.2 (0.5)	94.5 (0.5)	94.3 (0.9)
	Range	94.4–95.7	93.8–95.4	92.5–95.2
Reduced hydrolysed technetium (%)	Mean (SD)	0.8 (0.5)	0.7 (0.2)	0.7 (0.2)
	Range	0.4–1.7	0.4–1.0	0.4–1.0
Injected activity (MBq)	Mean (SD)	178.1 (4.8)	181.8 (5.1)	190.3 (8.0)
	Range	169–184	176–188	174–198
Volume administered (ml)	Mean (SD)	1.6 (0.1)	2.0 (0)	4.0 (0)
	Range	1.6–1.7	2.0–2.0	4.0–4.0

The injected radioactivity was similar across all dose groups, the mean injected activity being 183 ± 8 MBq (range: 169–198 MBq). One female participant received 1.25 ml (47 μg) of ^99m^Tc-maraciclatide instead of 2 ml (75 μg dose). As the radioactive dose was within the expected clinical range, data for this participant was included in the biodistribution and internal radiation assessment.

### Safety

^99m^Tc-maraciclatide was safe and well tolerated. There were no deaths, and no serious AEs (SAEs) were reported in the study (Table 7). Among the 24 participants receiving a protocol-stipulated dose of ^99m^Tc-maraciclatide, 17 (71%) participants experienced 31 non-serious AEs. However, only 7 (23%) were deemed by investigators to be related to maraciclatide, the majority of which were mild in intensity, and none resulted in participant withdrawal. Of the remaining AEs not attributed to maraciclatide, 10 were attributed to procedural complications, 5 were due to a pre-existing medical condition, and 6 were due to ‘other causes’ (including sleep deprivation and the effect of multiple blood draws). In the placebo group, 3 (38%) of the AEs were attributed to the placebo, 3 due to a procedural complication, 1 due to a pre-existing medical condition and 1 due to ‘other cause’ (lack of bowel movement).

**Table 7:** Recorded adverse events

MedDRA body system AE	15 μg maraciclatide (n = 8)			75 μg maraciclatide (n = 8)			150 μg maraciclatide (n = 8)			Placebo		
	No. of AEs	No. with AEs	Cause of AE	No. of AEs	No. with AEs	Cause of AE	No. of AEs	No. with AEs	Cause of AEs	No. of AEs	No. with AEs	Cause of AEs
Any system	12	6	-	9	5	-	10	6	-	8	4	-
GI disorder	2	1	-	0	0	-	1	1	-	2	2	-
Abdominal pain	1	1	IMP	-	-	-	1	1	Unrelated medical condition	-	-	-
Diarrhoea	1	1	IMP	-	-	-	0	0	-	2	2	Unrelated medical condition
Inx	2	2	-	5	4	-	3	3	-	3	3	
RBC decrease	-	-	-	2	2	Procedural complication	2	2	Procedural complication	1	1	Procedural complication
Haemoglobin decrease	0	0	-	1	1	Procedural complication	0	0	-	0	0	-
Eosinophilia	-	-	-	-	-	-	-	-	-	1	1	Unrelated medical condition
Blood creatinine phosphokinase increase	1	1	Other	0	0	-	0	0	-	0	0	-
Blood phosphate decrease	0	0	-	1	1	IMP	0	0	-	1	1	Unrelated medical condition
Bilirubin increase	0	0	-	0	0	-	0	0	-	0	0	Unrelated medical condition
Hyperkalaemia	0	0	-	0	0	-	1	1	Procedural complication	0	0	-
ECG T-wave inversion	1	1	Procedural complication	0	0	-	0	0	-	0	0	-
ECG QRS complex prolonged	0	0	-	1	1	Unrelated medical condition	0	0	-	0	0	-
ECG PR shortened	0	0	-	0	0	-	0	0	-	1	1	Unrelated medical condition
Crystal urine present	0	0	-	0	0	-	1	1	IMP	0	0	-
Skin disorder	1	1	-	0	0	-	0	0	-	1	1	-
Contact dermatitis	1	1	Procedural complication	-	-		-	-		1	1	Procedural complication
Vascular disorder	0	0	-	1	1	-	1	1	-	1	1	-
Haematoma	0	0	-	1	1	Procedural complication	1	1	Procedural complication	1	1	Procedural complication
Infection	1	1	-	0	0	-	1	1	-	0	0	-
Urinary tract infection	1	1	Unrelated medical condition	0	0	-	0	0	-	0	0	-
Upper respiratory tract infection	0	0	-	0	0	-	1	1	Unrelated medical condition	0	0	-
Herpes simplex virus	0	0	-	0	0	-	1	1	Unrelated medical condition	0	0	-
Cardiac disorder	0	0	-	1	1	-	0	0	-	0	0	-
Palpitations	0	0	-	1	1	Other	0	0	-	0	0	-
Musculoskeletal	0	0	-	1	1	-	1	1	-	0	0	-
Back pain	0	0	-	1	1	Procedural complication	1	1	Procedural complication	0	0	-
Nervous system	4	2	-	1	1	-	1	1	-	0	0	-
Headache	2	2	Unrelated medical condition	0	0	-	1	1	-	0	0	-
Hypoaesthesia	1	1	IMP	0	0	-	0	0	-	0	0	-
Dizziness	1	1	IMP	0	0	-	0	0	-	0	0	-
Hyporeflexia				1	1	Other	0	0	-	0	0	-
General disorders	2	2	-	1	1	-	0	0	-	0	0	-
Fatigue	1	1	Unrelated medical condition	1	1	Unrelated medical condition	0	0	-	0	0	-
Anorexia	1	1	Other	0	0	-	0	0	-	0	0	-

3 (13%) of the 24 participants who received ^99m^Tc-maraciclatide experienced AEs that required medical treatment. 1 in the 15 μg ^99m^Tc-maraciclatide group experienced contact dermatitis, so required Sudocrem, 1 received aciclovir for herpes simplex, and one received Soladepine for abdominal pain. None of these medical indications for treatment was attributed to ^99m^Tc-maraciclatide.

The most frequent AE was a reduction in red blood cell count, which occurred in 4 (17%) of the participants that received ^99m^Tc-maraciclatide, compared with 1 (13%) in the placebo group. The reduction in red cell count (RCC) was attributed to repeated blood samples taken in this study, there was no suggestion that this AE resulted from the marker. Other AEs that occurred more than once in the participants that received ^99m^Tc-maraciclatide were headache, fatigue, abdominal pain, and haematoma (each with 1–3 events and each occurring more frequently than in the placebo group). Furthermore, the laboratory, ECG and vital signs parameters displayed no clinically important safety trends and no apparent difference in the safety profile between dose groups and placebo was identified.

Of the participants who received ^99m^Tc-maraciclatide, 7 of the AE in 6 participants were attributed to the agent. Three were in the 15 µg dose group, one was in the 75 µg, and two were in the 150 µg group. All seven AEs deemed related to ^99m^Tc-maraciclatide were of mild intensity and resolved within 25 h.

### Biodistribution

The data acquired from all participants who received ^99m^Tc-maraciclatide within the expected clinical range (15 to 75 µg) were pooled as there were no obvious or clinically significant differences in the biodistribution data between dose groups or between male and female participants. The biodistribution data was determined as a percentage of injected activity.

**Fig. 2:**
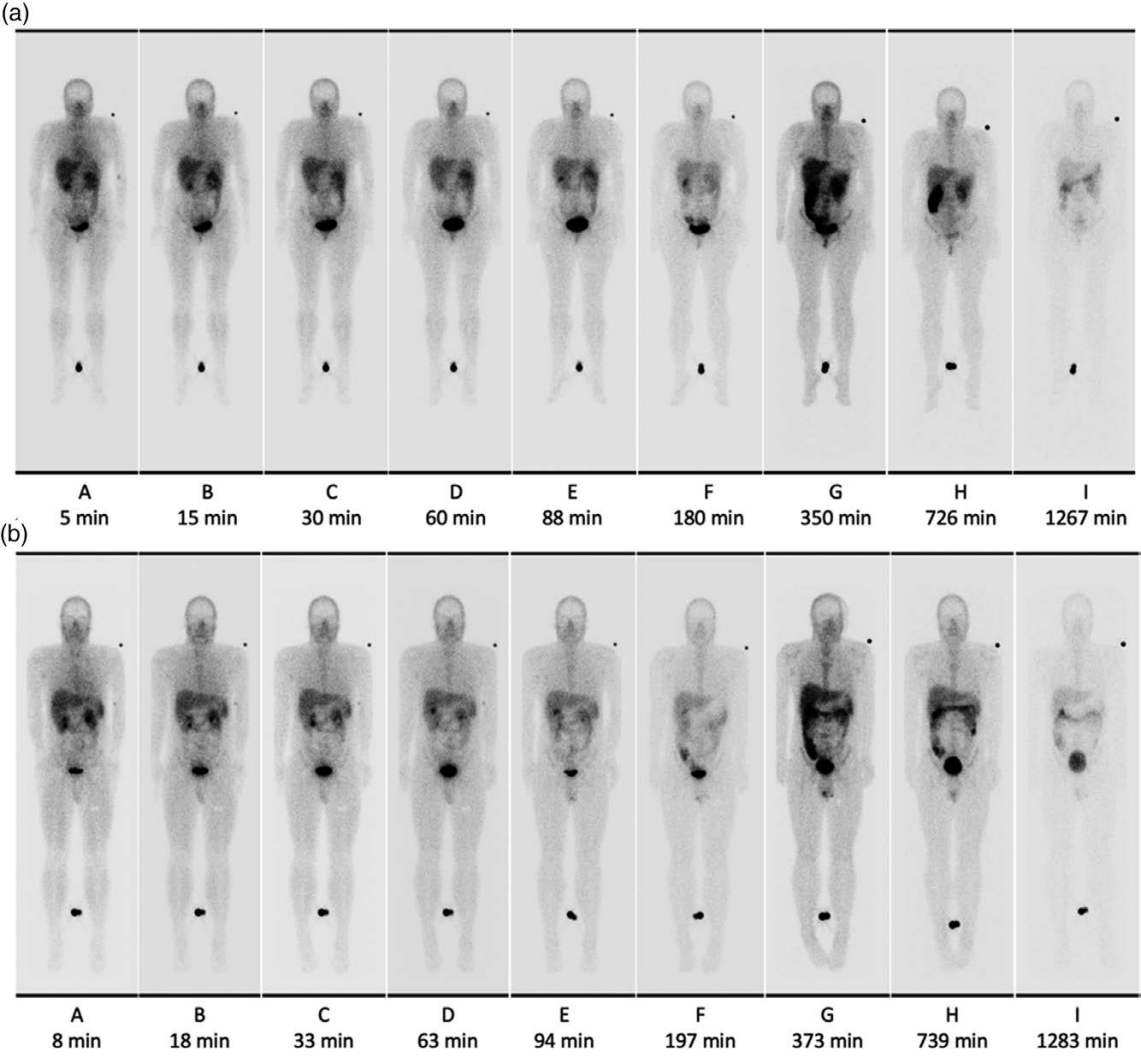
Whole body geometric mean emission images from one female (a) and one male (b) participant at multiple time points post-injection. The images were acquired for 6 min per scan at 5, 15, 30 min, 1 h, 1.5 h, and 3 h post-injection. Due to decay, the image acquisition was increased to 25 min per scan at 6 h and 35 min per scan at 12 and 24 h. Hence, the unadjusted images show differences in intensity across the timeline.

Following intravenous bolus administration of ^99m^Tc-maraciclatide, initial uptakes (nominally 5 min after administration) of ^99m^Tc were highest in the walls of the GI tract (20%), liver (15%), lungs (9%), kidneys (7%) and the contents of the urinary bladder (5%) (Fig. 2). Initially, the ROI set over the GI tract exhibited a rapid initial uptake of ^99m^Tc (especially in the first 15 min), predominantly within the luminal contents. However, it was possible to distinguish the ^99m^Tc in the luminal contents at later points. There was very low uptake in the salivary glands (0.7% at 5 min post-administration) in 15 participants and in the thyroid glands of four participants (0.5% at 5 min post-administration). Uptake in early images was observed in the red bone marrow (right and left ilia and spinal vertebrae); however, the uptake was too low to be quantified with any confidence.

The five regions with the highest normalised cumulated activities (excluding the remaining tissues category) were the contents of the urinary bladder and voided urine (3.4 ± 0.4 MBq*h/MBq), the combined walls of the small intestine and upper and lower large intestine (0.9 ± 0.2 MBq*h/MBq), liver (0.8 ± 0.2 MBq*h/MBq), lung (0.4 ± 0.1 MBq*h/MBq) and the contents of the upper large intestine (0.3 ± 0.1 MBq*h/MBq). The high value associated with the urinary bladder contents and voided urine reflects the high percentage of activity excreted via the urinary pathway. The correlation between the amount of radioactivity and maraciclatide measured indicated that the ^99m^Tc excreted was most likely as ^99m^Tc-maraciclatide [16]. This was further corroborated by the biodistribution not corresponding to that typically seen with free pertechnetate (high thyroid and salivary gland uptake) or RHT (high liver uptake).

**Fig. 3:**
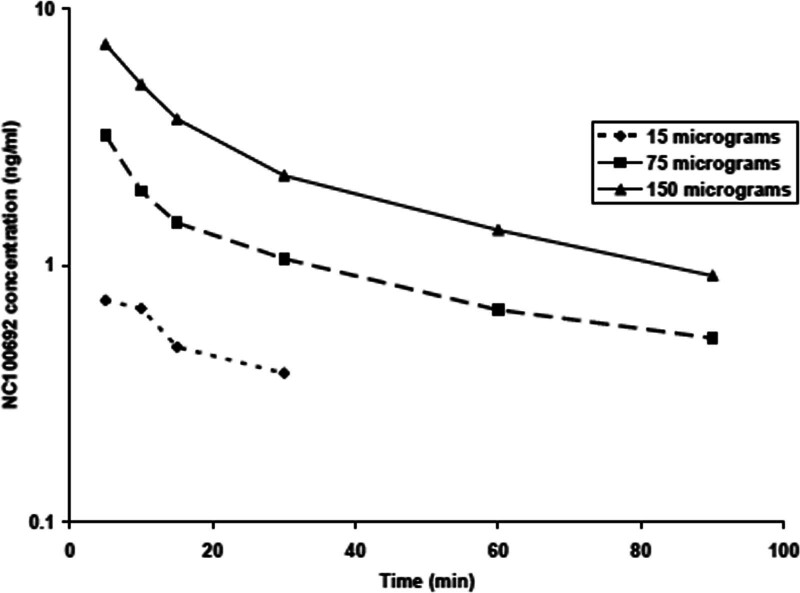
Plasma concentrations of maraciclatide following intravenous administration of ^99m^Tc-maraciclatide.

Plasma concentrations of maraciclatide exhibited a bi-exponential decline (Fig. 3) and were independent of the chemical dose. Concentrations of maraciclatide in plasma were assayed using the LC-MS method [14] (LOQ was 0.5 ng/ml). The mean apparent terminal elimination half-life was approximately 1 h. Overall, systemic clearance was approximately 6.7 ml/min/kg, whilst the mean apparent volume of distribution was approximately 520 ml/kg. The majority of ^99m^Tc activity was excreted through the urinary pathway and GI tract, 55% and 16%, respectively.

### Internal radiation dosimetry

The results of the organ & tissue dose calculations are given in Table 8.

**Table 8:** Organ absorbed dose per unit administered activity in normal human volunteers following an intravenous administration of ^99m^Tc-maraciclatide

Organ or tissue	Absorbed dose per unit administered activity [μGy/MBq]
Adrenal glands	4.7
Bone surfaces	4.5
Brain	1.3
Breasts	1.8
Gallbladder wall	8.7
Heart wall	6.1
Kidneys	16.5
Liver	11.1
Lower large intestine wall	11.7
Lungs	7.3
Muscle	2.8
Ovaries	6.3
Pancreas	5.0
Red marrow	3.0
Skin	1.5
Small intestine wall	8.9
Spleen	16.1
Stomach	3.8
Testes	2.8
Thymus gland	2.5
Thyroid gland	4.9
Upper large intestine wall	14.7
Urinary bladder wall	36.2
Uterus	7.5
Mean effective dose = 7.8 ± 0.8 μSv/MBq	

These data were calculated from the whole-body radioactivity as the cumulated activities from the biodistribution data, and the mean absorbed doses to 24 target organs were calculated using the Medical Internal Radiation Dosimetry (MIRD) schema [17]. The effective dose (ED) [18] was calculated from the ensemble of organ-absorbed dose values at 7.8 ± 0.8 μSv/MBq.

The five organs or tissues with the highest absorbed doses per unit injected activity were the urinary bladder wall (36.2 ± 7.0 μGy/MBq), kidneys (16.5 ± 9.5 μGy/MBq), spleen (16.1 ± 8.5 μGy/MBq), the wall of the upper large intestine (14.7 ± 4.0 μGy/MBq) and the wall of the lower large intestine (11.7 ± 2.7 μGy/MBq).

## Discussion

The aim of this study was to evaluate the clinical safety, internal radiation dosimetry and the whole-body biodistribution of ^99m^Tc-maraciclatide after intravenous bolus injection in healthy adult volunteers. This assessment of ^99m^Tc-maraciclatide is an essential prerequisite for its use as a potential radiopharmaceutical product for diagnostic imaging. The amounts of the chemical product selected for the study included the maximum expected clinical chemical dose (75 μg) and were evaluated up to twice this level. This was considered adequate to demonstrate the safety of ^99m^Tc-maraciclatide in routine clinical use. ^99m^Tc-maraciclatide was safe and well tolerated, with no deaths or SAEs reported in the study. The majority of reported AEs related to ^99m^Tc-maraciclatide were mild with a similar profile to placebo, and none resulted in participant withdrawals.

Following intravenous bolus administration of ^99m^Tc-maraciclatide, initial uptake was rapid, with excretion predominantly via the urinary pathway and GI tract. For a 740 MBq (20 mCi) administration, the ED of ^99m^Tc-maraciclatide is comparable to other scintigraphic imaging agents and radiological imaging modalities. However, the product was and still is investigational, and the reported data relates to dosimetry only and is not about product use.

The authors note that there was a significant delay between the completion of this clinical study and its publication. This was related to the early clinical development of ^99m^Tc-maraciclatide in oncology and cardiology, which was successful from an imaging perspective but did not provide useful additional clinical information that would change patient management [19,20]. ^99m^Tc-maraciclatide has yet to achieve its full clinical potential despite the potential range of diagnostic applications and its favourable safety profile. With the resurgence of clinical development in inflammatory arthritis, it is timely to have the dosimetry information published and available to support further clinical studies that may lead to maraciclatide becoming an established diagnostic radiopharmaceutical.

Since the clinical phase of this study was carried out, there have been advances in both imaging technology and software applications, which have improved the accuracy of dosimetry calculations. This has been particularly apparent with the recent increased interest in theragnostic and therapeutic radiopharmaceuticals. The advances in quantitative SPECT-CT for organ dosimetry, which is more accurate than planar imaging, and the use of imaging algorithms for edge detection and image data segmentation have all contributed to improvements in organ dosimetry. Nevertheless, these advances do not devalue the findings of the current study. The methods described are well-established and still applicable to many of the radiopharmaceutical products that are in widespread clinical use. We consider that the safety data and radiation dosimetry provided for ^99m^Tc-maraciclatide in this study is currently of great importance to the nuclear medicine community and will provide a sound basis for the future development and clinical application of the product.

### Conclusion

^99m^Tc-maraciclatide has been demonstrated to be safe and well tolerated in healthy male and female volunteers. The biodistribution data and radiation dosimetry calculations gave an ED similar to other ^99m^Tc agents used in nuclear imaging. These results encourage further development of ^99m^Tc-maraciclatide to identify its potential clinical use as an imaging agent.

## Acknowledgements

We acknowledge the contribution of Amersham Health Ltd with the study design and execution and thank Brian McParland, Heather Wray, Anne-Kirsti Aksnes and Nils Sponheim for their support.

This study was funded by Amersham Health Ltd.

### Conflicts of interest

Dr Gibbons’ Doctorate in Philosophy is part-funded by Serac Healthcare Ltd., Professor Perkins is a scientific advisor for Serac Healthcare Ltd, and Dr Jon Barnett is an employee of Serac Healthcare Ltd (^99m^Tc-maraciclatide).
